# Effect of Rocket (*Eruca sativa*) Extract on MRSA Growth and Proteome: Metabolic Adjustments in Plant-Based Media

**DOI:** 10.3389/fmicb.2017.00782

**Published:** 2017-05-05

**Authors:** Agapi I. Doulgeraki, Georgios Efthimiou, Spiros Paramithiotis, Katherine M. Pappas, Milton A. Typas, George-John Nychas

**Affiliations:** ^1^Laboratory of Microbiology and Biotechnology of Foods, Department of Food Science and Human Nutrition, Agricultural University of AthensAthens, Greece; ^2^Department of Genetics and Biotechnology, Faculty of Biology, School of Science, National and Kapodistrian University of AthensAthens, Greece; ^3^Laboratory of Food Quality Control and Hygiene, Department of Food Science and Human Nutrition, Agricultural University of AthensAthens, Greece

**Keywords:** MRSA, proteome, metabolism, defense, extract, rocket

## Abstract

The emergence of methicillin-resistant *Staphylococcus aureus* (MRSA) in food has provoked a great concern about the presence of MRSA in associated foodstuff. Although MRSA is often detected in various retailed meat products, it seems that food handlers are more strongly associated with this type of food contamination. Thus, it can be easily postulated that any food could be contaminated with this pathogen in an industrial environment or in household and cause food poisoning. To this direction, the effect of rocket *(Eruca sativa*) extract on MRSA growth and proteome was examined in the present study. This goal was achieved with the comparative study of the MRSA strain COL proteome, cultivated in rocket extract versus the standard Luria-Bertani growth medium. The obtained results showed that MRSA was able to grow in rocket extract. In addition, proteome analysis using 2-DE method showed that MRSA strain COL is taking advantage of the sugar-, lipid-, and vitamin-rich substrate in the liquid rocket extract, although its growth was delayed in rocket extract compared to Luria–Bertani medium. This work could initiate further research about bacterial metabolism in plant-based media and defense mechanisms against plant-derived antibacterials.

## Introduction

*Staphylococcus aureus* is an opportunistic pathogen bacterial species commonly found on the skin and hair, as well as in the respiratory tract of humans and animals. For a long period, it was recognized as a cause of nosocomial infection and since then it is known to acquire resistance to methicillin very rapidly. Strains of *S. aureus* that developed resistance to beta-lactam antibiotics -including penicillins, such as methicillin, dicloxacillin, nafcillin or oxacillin, and cephalosporins, through natural selection, are collectively referred to as methicillin-resistant *S. aureus* (MRSA). This hospital-focused epidemiological perception has recently changed due to the emergence of MRSA in food-producing animals, which has evoked a great concern regarding the presence of MRSA in associated foodstuff (Virgin et al., 2009; Ippolito et al., 2010). Earlier reports have clearly shown that food contamination by Staphylococci was due to the equipment and surfaces on which food is prepared (Gibson et al., 1999) and more recently, the presence of MRSA was confirmed in different retailed meat products (Kamal et al., 2013). However, the most common way for contamination of food with *Staphylococcus* is through contact with food workers who carry the bacteria or through contaminated milk and cheeses and/or contaminated food not properly refrigerated (Doulgeraki et al., 2017). Staphylococcal toxins can cause nausea, vomiting, stomach cramps, and diarrhea. The illness is usually mild and most patients recover after one to three days. In a small minority of patients, the illness may be more severe (Centers for Disease Control and Prevention [CDC], 2010).

*Staphylococcus aureus* strains have the ability to form multicellular communities that adhere on surfaces, i.e., biofilms (Christensen et al., 1994), a capacity that contributes significantly to antibiotic resistance (Gill et al., 2005). Biofilm-associated cells exhibit an altered phenotype with respect to bacterial physiology, metabolism and gene transcription (Donlan and Costerton, 2002). The availability of nutrients in a surface may also affect the attachment and biofilm formation of the pathogen and subsequently its phenotype. In a food industry environment, as well as in household during meal preparation, remaining nutrients on surfaces during the processing could favor the growth of microorganisms (Harris et al., 2003).

Salad rocket or arugula (*Eruca sativa*) is an edible plant that is commonly found in the market, often included in pre-cut salad packages. Rocket leaves, like many other plant leaves, contain glucosinolates (GSLs), a unique class of plant secondary metabolites (Antonious et al., 2009). When the leaves are cut or squashed during processing in the food industry, GSLs are released and hydrolysed, as they come in contact with the enzyme myrosinase, leading to the formation of several intermediate products, such as allyl isothiocyanates, methyl-isothiocyanates and 4-(methylsulfinyl)-butyl-isothiocyanate (Vig et al., 2009). Allyl isothiocyanates have been shown to exhibit antibacterial activity against *Bacillus cereus* IFO-13494, *B. subtilis* IFO-13722, *Escherichia coli* JCM-1649, *Pseudomonas aeruginosa* IFO-13275, *Salmonella* Enteritidis JCM-1891, *S. aureus* IFO-12732, *Vibrio parahaemolyticus* IFO-12711 (Isshiki et al., 1992), as well as *E. coli* O157:H7, *Listeria monocytogenes* (Lin et al., 2000), and *Helicobacter pylori* (Haristoy et al., 2005). Allyl isothiocyanate mode of action involves a change of bacterial membrane properties, decreasing bacterial surface charge and compromising the integrity of the cytoplasmatic membrane with consequent potassium leakage and propidium iodide uptake (Borges et al., 2015). Rocket extract also contains flavonoids, alkaloids and terpenoids that are known to have antimicrobial activity (Cowan, 1999; Hussein, 2013). Such plant secondary metabolites can cause the disruption of membrane function and structure (including the efflux system), interruption of DNA/RNA synthesis and function, interference with intermediary metabolism, induction of coagulation of cytoplasmic constituents and interruption of normal cell communication (Radulović et al., 2013).

However, in spite of the antimicrobials that salad rocket plants contain, *S. aureus* has been reported to contaminate rocket leaves and pre-cut salad preparations, to form biofilms and comprise a real risk for consumers’ health (Jahid and Ha, 2012; Baumgartner et al., 2014). Other bacterial pathogens, such as *Salmonella enterica* serovar Senftenberg and enterotoxigenic *E. coli* (ETEC), have been shown to attach on rocket leaves via their flagella (Berger et al., 2009; Shaw et al., 2011). However, as recently shown, the growth of *Salmonella enterica* serovar Typhimurium was limited on rocket leaves or in rocket extracts (Doulgeraki et al., 2016).

To our knowledge, few information are currently available about how MRSA grows into the juices of pre-cut rocket salads, defends against plant antimicrobials and exploits the nutrients present in plant-derived media. Therefore, the aim of this study was to monitor the effect of rocket extract on MRSA growth and proteomic profile. In order to achieve this, the MRSA strain COL was cultivated in liquid rocket extract and a standard complete growth medium (Luria–Bertani broth, LB) for comparison. The differentially expressed proteins in the rocket extract-grown cells were monitored using 2-DE analysis and bioinformatic tools based on 2D-gel map of the genomic *S. aureus* strain N315 for understanding the metabolic and molecular mechanisms that are active under these conditions.

## Materials and Methods

### Bacterial Strain and Growth Conditions

The MRSA *S. aureus* strain COL (kindly provided by Dr. Sophia Kathariou, North Karolina State University, USA) was used in this study. Cells in mid-exponential growth phase were harvested by centrifugation (5000 × *g*, 10 min, 4°C), washed twice with Ringer’s solution (1/4 strength that was used throughout) and finally resuspended in Ringer’s solution.

### Rocket Extract Preparation and Samples Preparation

Rocket leaves was purchased from a local supplier and rocket extract was prepared according to Doulgeraki et al. (2016). LB medium and rocket extract were inoculated with the appropriate inoculum in order to achieve a final population of 10–20 CFU/mL (Doulgeraki et al., 2016). The inoculated liquid cultures were incubated at 20°C and analyzed in duplicate at different time intervals. The nutrient composition of LB and rocket extract was calculated based on information supplied by the National Nutrient Database for Standard Reference Release 27, Agricultural Research Service, United States Department of Agriculture (USDA)^1^ Report No. 11959 (**Table 1**).

**Table 1:** Nutrient composition of Luria–Bertani (LB) and rocket extract.

	LB (g per 100 mL)	Rocket extract (g per 100 mL)^∗^
Total lipid	0.0045	0.66
Total sugar	0.008	2.05
Total protein	1.12	2.58
Vitamin C (ascorbate)	0	0.015
Vitamin B12 (cobalamin)	0.0000000025	0
Iron	0.0000202	0.00146

*^∗^Assuming that 100% of rocket nutrients passed to the extract (1:1 w/v).*

### Microbiological Analysis and Cell Collection

All samples were subjected to decimal serial dilutions in Ringer’s solution. Bacterial colonies were counted on LB agar plates after 18–24 h incubation at 37°C. For protein collection, appropriate volume of the liquid cultures (containing ∼10^8^ CFU) was centrifuged (5000 × *g*, 10 min, 4°C), the pellet was washed with phosphate-buffered saline (PBS) and stored at –80°C until further use.

### Protein Analysis

Sample preparation, protein quantification, isoelectric focusing in linear IPG 4–7 strip (17 cm, Bio-Rad Laboratories) and concomitant SDS-PAGE (12.5% T, 2.6% C) were performed according to previously described methods (Paramithiotis et al., 2013). The SDS-PAGE gels were scanned with a GS-800 Calibrated Densitometer (Bio-Rad Laboratories) and analyzed with PDQuest Advanced image analyser (Bio-Rad Laboratories), according to manufacturer’s instructions.

The differentially expressed proteins, appearing as spots on the gel were identified *in silico* with the proteomic tool SWISS-2DPAGE^2^. Since no *in silico* 2D-PAGE map for *S. aureus* COL is available yet, the Mw and pI coordinates of each spot were imported into the database designed from the genome of another MRSA *S. aureus* strain, N315 (Becker and Palsson, 2005; Fleury et al., 2009), and a tentative identification was obtained. The KEGG database for strain N135^3^ was then used for data interpretation.

The nucleotide sequences of homologue genes from the genome of *S. aureus* strain COL (Gill et al., 2005) and strain N315 were compared by using BLAST^4^ alignments and the % of identity verified the correct use of genes.

### Statistical Analysis

One-way analysis of variance (ANOVA) was used to statistically assess the differences between the *S. aureus* strain COL populations during growth in LB broth and rocket extract at 20°C.

## Results and Discussion

**FIGURE 1:**
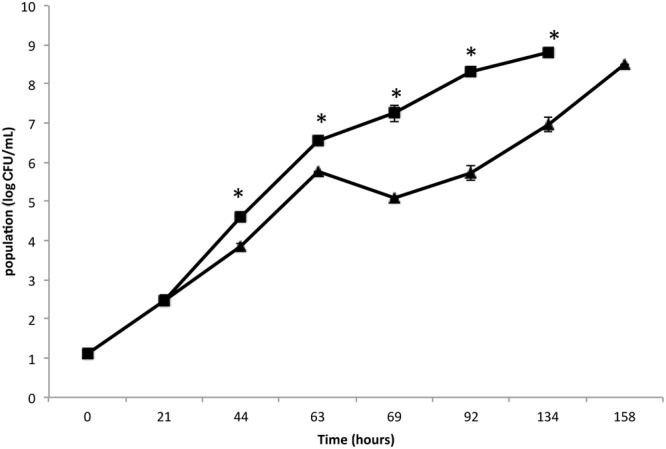
**Growth of *Staphylococcus aureus* strain COL on Luria–Bertani (LB) broth (▪) and rocket extract (▴) at 20°C.** Each number is the mean of two samples taken from different experiments. Each sample was analyzed in duplicate (coefficient of variation <5%). The bars represent the standard deviations. The asterisk indicates statistically significant differences (*P* < 0.05).

The present study aimed to better understand the behavior of an MRSA *S. aureus* strain on leafy salads, such as rocket, simulating a scenario in which the pathogen survives, adheres and forms biofilm by exploiting nutrient leftovers produced during the slicing/damage of plants in an industrial environment or household. Initially, the growth of MRSA strain COL was monitored and compared in rocket extract and LB (**Figure 1**). The results clearly indicate that MRSA is capable of growing in rocket extract, although at slower rates, reaching after 7 days the same cell concentration as that of LB culture at 4 days. This may be due to the presence of inhibitory compounds such as flavonoids, alkaloids and terpenoids (Cowan, 1999; Hussein, 2013). Similar growth inhibition of microorganisms by plant extracts has been previously reported (Abdou et al., 1972; Tiedink et al., 1991; Vig et al., 2009; Doulgeraki et al., 2016). The fact that MRSA managed to grow in rocket extract, eventually reaching similar growth levels with these of the LB culture, indicates that, over time, it manages to overcome the early negative effect of the plant extract. In this respect, it is interesting to note that both probiotic bacteria *Lactobacillus acidophilus* DSM 20079 and *L. plantarum* DSM 20174 grew more efficiently in a rocket extract-based medium and that their antioxidant and antimicrobial power was significantly increased (Fratianni et al., 2014).

**FIGURE 2:**
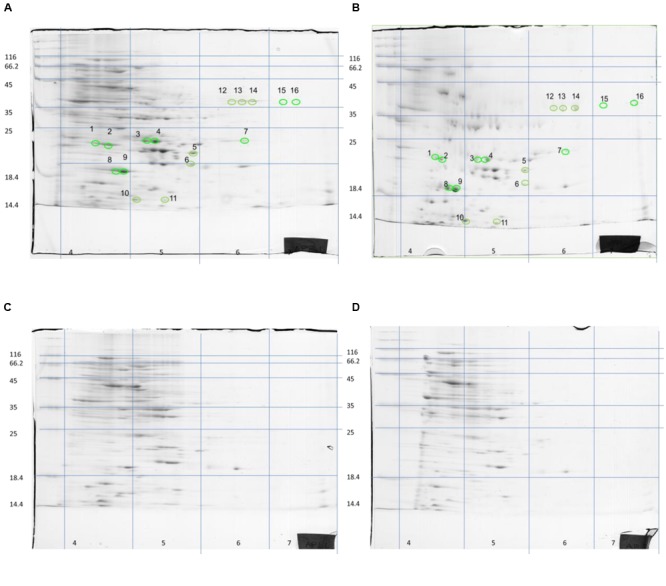
**Two-dimensional separation of total protein extracted from *S. aureus* COL cells growing in liquid rocket extract (A,B)** or LB **(C,D)**. Circles and numbers correspond to the protein spots listed in **Table 2**.

Although *S. aureus* strain COL has been reported to code for 2,676 putative ORFs (Gill et al., 2005), only a few hundred spots were visible on the gels of this study. However, this is not uncommon as similar technology cut-off limitations have been encountered by previous researchers in their attempts to reconstruct *in silico* the metabolic pathway of the bacterium at genome-scale, where only the 23% of the ORFs could be determined (Heinemann et al., 2005). Nevertheless, the present proteome analysis as shown by the separation of staphylococcal proteins after 2D-gel electrophoresis revealed a number of protein spots that were differentially expressed in the two media. In a similar manner, *L. acidophilus* DSM 20079 and *L. plantarum* DSM 20174 over-expressed a small number of proteins when grown in rocket salad extracts as judged by the electrophoregrams of on-chip microelectrophoresis (Fratianni et al., 2014). The location of the differentially expressed proteins that have been observed on the polyacrylamide gels is shown in **Figure 2**, and further details for each spot are provided in **Table 2**. Overall, 13 proteins were expressed as distinct spots only by the rocket extract-grown staphylococci, being always absent from the protein expression profiles of the LB-grown cells (namely, #1,3-7; #10-16). In addition, three more spots (#2,8,9) were significantly over-expressed in the rocket extract-grown cells in comparison with those obtained from the LB-grown cultures.

**Table 2:** Details of the 16 differentially expressed protein spots observed on the 2D-gels.

		Approximate range of Mw and pI coordinates for each spot			
		Mw (kDa)		pI	
Spot #	Presence/absence or over-expression	min	max	min	max
1	Pr	21.5	23.5	4.4	4.6
2	O	21.5	23.5	4.4	4.6
3	Pr	22.5	24.5	5.2	5.4
4	Pr	22.5	24.5	5.3	5.5
5	Pr	20.5	22.5	5.8	6.0
6	Pr	19.5	21.5	5.7	5.9
7	Pr	22.0	24.0	6.5	6.7
8	O	18.4	18.6	4.7	4.9
9	O	18.4	18.6	4.8	5.0
10	Pr	14.5	16.5	5.0	5.2
11	Pr	14.5	16.5	5.5	5.7
12	Pr	35.0	37.0	6.3	6.5
13	Pr	35.0	37.0	6.5	6.7
14	Pr	35.0	37.0	6.6	6.8
15	Pr	35.0	37.0	7.2	7.4
16	Pr	35.0	37.0	7.3	7.5

*The ranges of Mw and pI values were imported into the SWISS-2DPAGE database for further study. Pr: present only in rocket-extract, O: over-expressed in rocket-extract grown cells.*

To identify the 16 spots, the well-established proteomic tool SWISS-2DPAGE^2^ was employed. This tool provides a well-characterized proteomic profile (2D-gel map) based on the genome of another MRSA *S. aureus* strain N315 (Fleury et al., 2009). The pI and Mw values of these differentially expressed spots were imported into the database (at a range of 0.2 units) and a tentative identity for a number of these spots was obtained (**Table 3**). Six proteins that were present in the rocket extract-grown cells but absent in the LB-grown cultures were identified (#1,3,4,5,6,10). For the remaining 10 spots, no clear identities were provided by the database. More specifically, protein #1 corresponded to a 3-hexulose-6-phosphate synthase (EC = 4.1.2.43), a ribulose monophosphate pathway enzyme involved in formaldehyde fixation and detoxification. Protein #3, present with two gene copies in both N315 and COL (**Table 3**), was a 3-oxoacyl-[acyl-carrier-protein] reductase (EC = 1.1.1.100), involved in fatty acid biosynthesis and biotin metabolism. Proteins #5 and #6 were a peptide deformylase (PDF; EC = 3.5.1.88) and a FMN-dependent NADPH-azoreductase (EC = 1.7.–.–), respectively, with a yet unknown pathway involvement for both. Protein #10 was a nucleoside diphosphate kinase (NDK) (EC = 2.7.4.6), with a possible involvement in purine/pyrimidine synthesis and synthesis of secondary metabolites. Finally, protein #4, a nitroreductase, corresponded to two different nucleotide sequences in N315 (gene SA1840 and SARS04315) and COL (SACOLRS10550 and SACOL0874, respectively) each of which showed a 99% nucleotide sequence identity with its homologue gene, but in reverse-strands. Thus, it appears that the two strains also have two copies of nitroreductase, but the homologues are located in opposite strands. All homologue genes in *S. aureus* COL and N135 had 99-100% nucleotide sequence identities (**Table 3**).

**Table 3:** Details of the most distinct differentially expressed protein spots and involvement of the identified proteins in specific metabolic pathways.

	Swiss-2DPAGE					
Spot #	ID code	ID name	Mw (Da)	pI	Homologue genes in COL and N315 strains	Pathway
1	Q7A774	3-hexulose-6-phosphate synthase	23438	4.50	SACOL0617	Pentose phosphate pathway; Methane metabolism
3	P99093	3-oxoacyl-[acyl-carrier-protein] reductase FabG	23228	5.34	SACOL1245; SACOL1299	Biosynthesis of unsaturated fatty acids; Fatty acid biosynthesis; Biotin metabolism
4	Q7A4J0	Putative uncharacterized protein SA1840	24186	5.44	SACOL0874	Nitroreductase
5	P99077	Peptide deformylase	21683	5.87	SACOL1100; SACOL1227	–
6	Q7A782	FMN-dependent NADPH-azoreductase	19732	5.76	SACOL0190	–
10	P99068	Nucleotide diphosphate kinase	14586	5.04	SACOL1509	Purine and pyrimidine synthesis; Synthesis of secondary metabolites

*In all cases, COL and N315 homolog genes had 99–100% identity.*

As mentioned above, 3-hexulose-6-phosphate synthase (HPS, spot #1), is involved in formaldehyde fixation and detoxification. This may initially appear surprising because the enzyme is mainly present in microorganisms that are capable of utilizing C1-compounds, but it is now recognized as involved in alternative metabolic functions, i.e., the catalysis of the reverse reaction for the biosynthesis of pentose phosphate in several bacteria (Behr et al., 2007). Its presence in the genomes of human pathogenic bacteria like *Salmonella enterica* serovar Typhi, *S. enterica* serovar Typhimurium, *E. coli, S. aureus*, and *S. epidermitis*, as well as in *Lactobacillus brevis* and *L. casei*, which are known as beer spoilage bacteria, indicates a more widespread presence and multifunctional role of HPS. It may, therefore, be postulated that the significant over-expression of HPS observed in these experiments is due to the utilization by the COL strain of carbohydrates found in the rocket extract via the pentose phosphate pathway, in order to satisfy its cellular needs. Interestingly, different plants (vegetables, fruits, especially in red beet, cauliflower, kohlrabi, grapes) contain a large amount of releasable endogenous formaldehyde (0.5–1.0 mM) bound to L-arginine mainly in the form of N(G)-trihydroxymethyl-L-arginine (TriHMA) (Trézl et al., 1998). Thus, the proteomic approach for the characterization of hop-inducible proteins in *L. brevis* that recorded an over-expression of HPS in bacteria grown in plant extract medium (Behr et al., 2007) comes in support of our results and strengthens the suggestions for a multifunctional role of this enzyme.

From the uniquely expressed enzymes only in rocket extract, 3-oxoacyl-[acyl-carrier-protein] reductase (FabG, spot #3), has a conserved NAD(P) binding domain, and is involved in the biosynthesis of fatty and unsaturated fatty acids. It catalyses the reversible reduction of (R)-3-hydroxy-enoyl-ACP to (R)-3-oxo-enoyl-ACP, which is an intermediate reaction for the synthesis of saturated or unsaturated fatty acids. Since the extract medium is richer in lipids and carbohydrates that are subsequently converted to fatty acids (**Table 1**), it is possible that this pathway is more active in the extract-grown cells. In support of this hypothesis come the inhibitory effects that maple leaf extracts and tannic acid had on *S. aureus, S. epidermidis, S. enterica*, and many species of *Shigella, Klebsiella* and other human pathogenic bacteria used to evaluate FabG as a possible antibacterial target (Wu et al., 2010). FabG is also involved in the biosynthesis of biotin, a vitamin that is an essential cofactor in carboxylation, decarboxylation, and transcarboxylation reactions and in particular in fatty acid synthesis, branched-chain amino acid catabolism, and gluconeogenesis (Streit and Entcheva, 2002). Thus, it is interesting to notice that although amounts of biotin are synthesized via the conversion of pimelate and there is no need for biotin to be added in a minimal medium for the growth of *S. aureus* COL, ORFs for biotin accessory proteins, such as BioH, BioC, and BioY are absent from the genome of the bacterium (Lee et al., 2009).

Concerning the three enzymes that were identified but their pathway involvement was less clear, they included: a PDF (EC = 3.5.1.88) that acts on carbon-nitrogen bonds (PDF, spot #5), a nitroreductase that reduces nitrogen-containing compounds (NfrA, spot #4), and an FMN-dependent NADPH-azoreductase (EC = 1.7.–.–) that acts on nitrogenous compounds (Azo1, spot #6). The metalloproteinase PDF has a very important role in bacteria in removing the *N*-formyl group from the terminal methionine residue of nascent proteins, a necessary step to complete protein biosynthesis and maturation. The crystal structure of *S. aureus* PDF has been studied in complex with the naturally occurring antibacterial agent actinonin, and it has revealed its high potential to be used as a specific target for the discovery of broad spectrum antibacterial drugs (Yoon et al., 2004). The nitroreductase enzyme in spot#4 corresponded to the studied NfrA protein of *S. aureus* (Streker et al., 2005), which was found capable of reducing FMN in the presence of NADPH, reducing organic nitro compounds like nitrofurantoin and nitrofurazone, and exhibiting weak disulfide reductase activity. It was suggested that NfrA contributes electrons from NADPH to different oxidized or otherwise damaged proteins under different stress conditions in *S. aureus*. Since some plants like asparagus are known to synthesize nitroglycosides as defense mechanism against plant pathogenic bacteria (Anderson et al., 1993), the unique presence of this enzyme in rocket extract may be explained as a putative counter action/defense response of *S. aureus* COL to similar substances that may be contained in the extract. The (FMN)-dependent NADPH-azoreductase (Azo1) identified from spot #6 is an enzyme previously biochemically and molecularly characterized from *S. aureus* as a tetrameric NADPH-dependent flavoprotein capable of cleaving a number of model azo dyes (Chen et al., 2005). In *Salmonella*, the same enzyme -coded by gene *azo*R- was up-regulated in cilantro and lettuce. As the enzyme is connected with cleavage of aromatic azo compounds, it may be involved in the defense of the bacterium against plant antibacterial compounds of aromatic structure (Goudeau et al., 2013). Azoreductases are also known to reduce azo-dyes that are extensively sprayed as color additives on vegetables or other food products. Bacteria of the intestinal microbiota produce azoreductases and reduce ingested azo-dyes (Rafii et al., 1990), a function that the *S. aureus* strain ATCC 25923 isolated from human skin was reported to have (Chen et al., 2005). It is not unlikely that traces of azo-dyes were present on the rocket leaves used in this study. Azoreductase was also over-expressed in *E. coli* O157:H7 EDL933 (EHEC) cells growing on radish sprouts leading the authors to the conclusion that azoreductase plays a role in detoxification against plant-derived antimicrobial agents (Landstorfer et al., 2014). However, it must be pointed out that this was not observed when the same bacterium grew in spinach juice.

The enzyme corresponding to spot #10, NDK, is an essential for the cell enzyme that plays a major role in the synthesis of nucleoside triphosphates (NTPs) and nucleoside diphosphates (NDP) which maintain nucleotide pools in the cell. Although NDK proteins have moderately similar sequence identities, they are highly conserved in prokaryotes and eukaryotes performing similar functions (Sundin et al., 1996). In bacteria, extracellular secretion of NDK has been reported in several pathogens such as *Porphyromonas gingivalis* (Yilmaz et al., 2008), *Pseudomonas aeruginosa, Mycobacterium tuberculosis* (Chakrabarty, 1998), and *S. enterica* serovar Typhimurium (Dar et al., 2011). It appears that apart from its major role in supporting growth and colonization of bacteria, NDK takes also part in other functions like virulence, cell signaling and polysaccharide synthesis, as demonstrated in *Mycobacterium* and *Pseudomonas* species (Chakrabarty, 1998). NDK catalyzes the formation of GTP which is a precursor of ppGpp, and this global regulatory molecule acts as a signal transduction molecule and mediates environmental regulation of both invasion and intracellular virulence genes in *Salmonella* (Thomson et al., 2006). GTP is also a precursor of GDP-mannose, an intermediate in alginate synthesis, and as an extracellular polysaccharide alginate introduces the mucoidal growth of bacteria like *Pseudomonas* and *Mycobacterium*, a type of growth directly linked with reduced secretion of virulence factors (Chakrabarty, 1998). In addition, NDK is involved in the formation of multi-enzyme complexes in *E. coli* (Mathews, 1993) and the enzyme produced by *Mycobacterium* was shown to bind to and inactivate the small GTPase Rac1 in the macrophages, thus causing a defect of both NOX2 assembly and production of reactive oxygen species (ROS) in response to wild type pathogen (Sun et al., 2013). It is noted that NDK-complexing proteins from prokaryotes can form complexes with human NDK to generate specific NTPs, and that some mammalian regulatory proteins can form complexes with bacterial NDKs to generate GTP (Shankar et al., 1997). Recently, the proteomic analysis by 2D-gel electrophoresis and mass spectrometry of the causal agent of bacterial cancer *Clavibacter michiganensis* subsp. *michiganensis* growing on *Lycopersicon hirsutum*, has shown that NDK was also over-produced by the bacterium (Afroz et al., 2013).

In general, it is expected that human pathogens will find it difficult to grow on plant tissues where growth nutrients will be less abundant. However, fresh vegetables have been recurrently associated with salmonellosis outbreaks (Herman et al., 2015) and both *S. enterica* and *E. coli* serovar O157:H7 can multiply on cilantro and lettuce if they have access to plenty of free water and optimal temperature conditions (Brandl and Amundson, 2008; Goudeau et al., 2013). They may even thrive if the plant tissues are previously mechanically damaged or infected by bacterial plant pathogens so that released plant cell contents provide substrates for growth to the human pathogenic bacteria (Brandl, 2008). In that sense, it is very interesting to observe that the enzymes that have been detected to be over-expressed in rocket extracts are similar to enzymes that were up-regulated in *Salmonella* that grew on lettuce (Goudeau et al., 2013). This indicates that human pathogenic bacteria adapt quickly to the changing conditions by enhanced expression of a certain subset of genes and that their metabolism in plant-based media or surfaces is somehow conserved, characterized by a response to the different media composition (Goudeau et al., 2013). A more recent proteomic study of *Salmonella* protein expression inside lettuce leaves in which the results were similar with the above further supports this hypothesis (Zhang et al., 2014).

Although, the present results are preliminary and cannot suggest a defense mechanism against plant-derived antimicrobials, such a reaction would hypothetically explain how MRSA managed to overcome the early stage inhibition in liquid rocket extract and grow to similar levels with the LB culture after 7 days. The over-expression of azoreductase that was also observed in plant-growing *Salmonella* and *E. coli* cells, as mentioned above, could be a detoxification mechanism. However, to establish such a hypothesis further research is needed.

Overall, the present findings indicate that *S. aureus* COL is growing satisfactorily in the liquid rocket extract, taking advantage of the sugar-, lipid- and vitamin-rich substrate and overcoming the early growth inhibition effect. This is very important for the food industry, as it shows that MRSA is tolerant to the plant secondary metabolites with antimicrobial activity that are released when rocket is cut or squashed and can therefore grow in pre-cut rocket salad preparations or related surfaces, especially if left at room temperature for 1–2 days or longer. This work could initiate further research regarding bacterial metabolism in plant-based media and defense mechanisms against plant-derived antibacterials. Beyond the overall findings described above, the most important one is the very fact that an important pathogen like MRSA is able to survive, grow and adapt in the food sector. Prioritizing the research in understanding the behavior of antibiotic resistant bacteria in food matrices, will enable novel strategies and treatments development for elimination of pathogenic bacteria in food chain and subsequently for consumer protection.

## Author Contributions

Conceived and designed the experiments: AD, G-JN. Performed the experiments: AD, SP. Analyzed the data: AD, GE. Wrote the manuscript: AD, GE, SP, KP, MT, G-JN.

## Conflict of Interest Statement

The authors declare that the research was conducted in the absence of any commercial or financial relationships that could be construed as a potential conflict of interest.

## Footnotes

1 http://ndb.nal.usda.gov/ndb/foods

2 http://world-2dpage.expasy.org/swiss-2dpage/

3 www.kegg.jp

4 http://blast.ncbi.nlm.nih.gov/Blast.cgi
